# Side Effects of Immunosuppressant Drugs After Liver Transplant

**DOI:** 10.3390/ph18030342

**Published:** 2025-02-27

**Authors:** Filippo Gabrielli, Elisa Bernasconi, Arianna Toscano, Alessandra Avossa, Alessia Cavicchioli, Pietro Andreone, Stefano Gitto

**Affiliations:** 1 Internal and Metabolic Medicine, Department of Medical and Surgical Sciences for Children & Adults, AOU of Modena, University of Modena and Reggio Emilia, 41126 Modena, Italy; 2 Postgraduate School of Internal Medicine, University of Modena and Reggio Emilia, 41126 Modena, Italy; 3 Division of Internal Medicine, University Hospital of Policlinico G. Martino, 98124 Messina, Italy; 4 Department of Experimental and Clinical Medicine, University of Florence, 50134 Florence, Italy; 5 Postgraduate School of Allergology and Clinical Immunology, University of Modena and Reggio Emilia, 41126 Modena, Italy

**Keywords:** orthotopic liver transplant, immunosuppression, side effect, cardiovascular disease, nephrotoxicity, metabolic syndrome, MASLD, osteoporosis, tacrolimus, frailty

## Abstract

Liver transplantation (LT) is the standard of care for both end-stage liver failure and hepatocellular carcinoma (HCC). Side effects of the main used immunosuppressive drugs have a noteworthy impact on the long-term outcome of LT recipients. Consequently, to achieve a balance between optimal immunosuppression and minimal side effects is a cornerstone of the post-LT period. Today, there are no validated markers for overimmunosuppression and underimmunosuppression, only a few drugs have therapeutic drug monitoring, and immunosuppression regimens vary from center to center and from country to country. Currently, there are many drugs with different efficacy and safety profiles. Using different agents permits a decrease in the dosage and minimizes the toxicities. A small subset of recipients achieves immunotolerance with the chance to stop immunosuppressive therapy. This article focuses on the side effects of immunosuppressive drugs, which significantly impact long-term outcomes for LT recipients. The primary aim is to highlight the balance between achieving effective immunosuppression and minimizing adverse effects, emphasizing the role of personalized therapeutic strategies. Moreover, this review evaluates the mechanisms of action and specific complications associated with immunosuppressive agents. Finally, special attention is given to strategies for reducing immunosuppressive burdens, improving patient quality of life, and identifying immunotolerant individuals.

## 1. Introduction

Liver transplantation (LT) represents the standard of care for end-stage liver diseases and hepatocellular carcinoma (HCC). Significant advancements in surgical techniques and the introduction of novel immunosuppressive drugs with tailored therapeutic protocols have substantially improved short-term survival in LT recipients, with a low rate of organ rejection (1.7%) [1] and a 10-year survival rate ranging from 61% to 65% [1,2]. The main causes of mortality are graft failure, infections, metabolic and cardiovascular (CV) complications, and malignancy [3]. Notably, immunosuppression can be partially or entirely responsible for the development of de novo malignancies, graft failure, or infectious risk, with an almost direct association. Conversely, in case of CV and metabolic complications, such as arterial hypertension, insulin resistance or type 2 diabetes mellitus (T2DM), overweight/obesity, dyslipidemia, and more generally, the development of metabolic syndrome (MetS), the role of immunosuppressive drugs—discussed in detail later—can contribute to the de novo onset of these conditions or exacerbate pre-existing ones. It is important to emphasize that recent decades have witnessed a significant increase in the prevalence of obesity [4], T2DM [5], and MetS [6] in the general population to the extent that they are now described as an epidemic. This trend has led to a rise in CV diseases both in the general population and among patients with liver disease. Furthermore, due to advancements in antiviral therapies that have resulted in the eradication of hepatitis C virus and the improved management of hepatitis B virus infection, Metabolic Associated Steatohepatitis (MASH) has become the second-leading cause of placement on the waiting list for LT in the United States [7]. In 2021, 19.3% of patients on the waiting list for LT in the United States had liver disease related to MASH. Moreover, in certain subgroups, such as patients over 65 years of age, MASH was the primary indication for transplantation [8]. Fibrosis in patients with MASH has been demonstrated to be the most important predictor of mortality from both hepatic and extrahepatic causes. Among the extrahepatic causes of death in MASH, CV diseases and extrahepatic malignancies represent the leading causes of mortality [9,10,11,12,13,14].

Therefore, although some cardiometabolic risk factors may be induced de novo by immunosuppression, a significant proportion of patients on the waiting list for LT already present pre-existing cardiometabolic conditions, which can be exacerbated by immunosuppressive therapy. Selecting the most appropriate drug to address the oncological, infectious, cardiometabolic, nephrotoxic, neurotoxic, and myelotoxic complications of LT patients is therefore crucial, emphasizing the need to tailor immunosuppressive therapy to the individual.

This review evaluates the side effects of immunosuppressive drugs used in LT, detailing the risk and underlying mechanisms associated with each drug class in the development of specific complications.

## 2. Methods

We developed a non-systematic review using the following electronic sources: PubMed, MEDLINE, Google Scholar, Ovid, Scopus, and Web of Science. We used the following search words: “LT”, “immunosuppression”, “basiliximab”, “corticosteroid”, “cyclosporine”, “tacrolimus”, “mycophenolate”, “azathioprine”, “sirolimus”, and “everolimus” alone or in combination with “cardiovascular disease”, “liver transplantation”, “epidemiology”, “neoplasm”, “cancer”, “metabolic syndrome”, “side effect”, “infection”, “graft survival”, “nephrotoxicity”, “neurotoxicity”, “myelosuppression”, “osteoporosis”, “frailty”. We considered all papers reporting human-related data (inclusion criteria), excluding articles with unavailable full text, not in the English language, abstracts, book chapters, and articles published before 1990 (exclusion criteria). We then examined supplementary references/articles among manuscripts considered in the first research round.

## 3. Immunosuppression After LT: The State of the Art

Notably, the liver is a privileged organ in terms of immunotolerance. Indeed, simpler and lower-dose regimens are necessary, especially in the long-term period, in comparison to other solid organ transplants [15,16].

In clinical practice, immunosuppression after LT is divided into two phases: an induction phase and a maintenance phase. The induction phase is initiated immediately after surgery to prevent rejection and immune-mediated graft damage. Current European and American guidelines [15,17] recommend a triple-drug immunosuppressant regimen consisting of CNIs (preferably TAC over CsA), a short course corticosteroid (3 months), and an antimetabolite (preferably MMF over AZA) as the primary choice to ensure graft survival while minimizing the side effects associated with these drug classes. Alternative drug combinations, including antibodies such as basiliximab, may also be used in patients who require a steroid-sparing or CNI-sparing regimen [18]. The maintenance regimen is initiated 30 days after transplantation, and in the majority of LT recipients, immunosuppression is continued lifelong. This regimen is individualized based on the patient’s comorbidities, the recurrence risk of the pretransplant condition (especially hepatitis C or autoimmune liver disease), the risk of infection or malignancy, and the potential toxicities of the medications. In patients with non-melanoma skin cancer, the use of everolimus (EVR), initiated one week after transplantation, or sirolimus (SRL), started one month post-transplant, is recommended [15]. Some evidence has also shown that immunosuppressive regimens incorporating mTOR inhibitors may reduce the recurrence of HCC, although specific randomized controlled trials (RCTs) have not yet been conducted [19,20,21,22,23].

## 4. Mechanism of Action of Immunosuppressant Drugs

**Figure 1:**
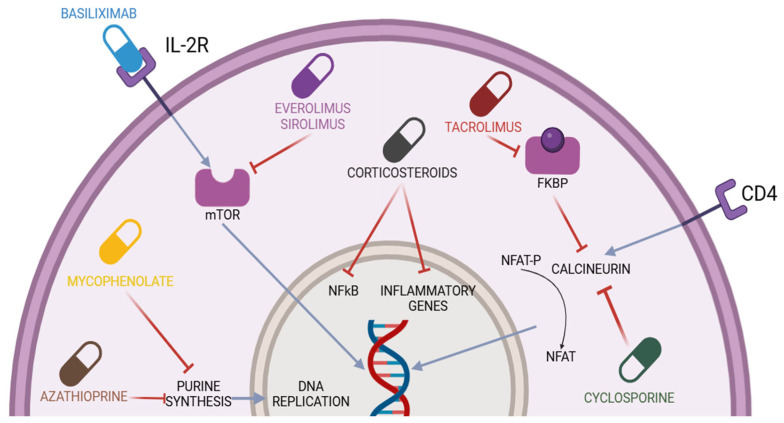
Immunosuppressive drugs used in liver transplantation and their mechanisms of action. CD4 = cluster differentiation 4; NFAT-P = nuclear factor of activated T-cell precursor; NFAT = nuclear factor of activated T-cells; IL-2R = interleukin-2 receptor; FKBP = FK506 binding proteins; NF-kB = nuclear factor kappa-light-chain-enhancer of activated B-cells; mTOR= mammalian target of rapamycin. The blue lines represent activation mechanisms, while the red lines represent inhibition mechanisms.

This section will address the mechanisms of action of the main immunosuppressive drugs used in LT. The immunosuppressive drugs typically used in LT belong to the class of calcineurin inhibitors (CNIs), including cyclosporine (CsA) and tacrolimus (TAC), corticosteroids (primarily prednisone), antimetabolite drugs (mycophenolate mofetil (MMF) and azathioprine (AZA)), inhibitors of the mammalian target of rapamycin (mTOR-I, everolimus (EVR), and sirolimus (SRL)), and biologic agents (basiliximab). Figure 1 shows the various molecules used in the context of post-LT immunosuppression and their respective mechanisms of action.

### 4.1. Calcineurin Inhibitors (CNIs)

CNIs are the backbone of most post-LT immunosuppression regimens; it has been estimated that 97% of patients discharged after an LT are on a CNI-based therapy [24]. Tacrolimus (FK506) was isolated from the soil fungus *Streptomyces tsukubaensis* [25]. Physiologically, calcineurin is a serine/threonine phosphatase composed of a catalytic subunit that binds to calmodulin (calcineurin A) and a regulatory subunit that can bind intracellular calcium (calcineurin B), thereby regulating the activity of calcineurin A [26,27]. At the level of T lymphocytes, the binding between the T-cell receptor (TCR) and an antigen leads to an increase in intracellular calcium concentrations, which activates calcineurin B. As a result, calcineurin A exerts its enzymatic activity and dephosphorylates the nuclear factor of activated T-cells (NFATs). At this point, both calcineurin A and NFATs are able to translocate into the nucleus, where they are involved in the transcriptional activation of genes encoding various cytokines, particularly interleukin-2 (IL-2) [28]. IL-2 is responsible for the growth and differentiation of T lymphocytes. CsA and TAC inhibit calcineurin by binding to intracellular small molecules: cyclophilin for CsA and FKBP12 for TAC. These are low-molecular-weight intracellular proteins that facilitate protein folding, intracellular protein transport, and the stability of multiprotein complexes [18]. While some side effects, such as alopecia, gingival hypertrophy, and hirsutism, may be tolerated by patients, others, such as hypertension, de novo diabetes, and hyperlipidemia, can significantly impact long-term outcomes [29]. However, the greatest concerns are related to the potential nephrotoxicity and neurotoxicity of the drugs.

### 4.2. Corticosteroids

Corticosteroids are used for the induction and maintenance of immunosuppression and treating acute cellular rejection.

Corticosteroids are steroid hormones, small lipophilic molecules capable of crossing the cell membrane. They exert their effects in different ways depending on the dosage [30]. In the case of low doses (e.g., <30 mg of prednisone), the action is primarily genomic. In this case, an intracellular interaction occurs between the corticosteroid and the cytoplasmic glucocorticoid receptor (GR) [31]. Once this binding occurs, the GR undergoes hyperphosphorylation, enabling it to migrate into the nucleus, where it regulates gene expression. This action can directly affect certain genes but also, and perhaps more importantly, influence various transcription factors, including NF-kB and AP-1. Moreover, the action extends to genes encoding enzymes involved in gluconeogenesis, such as tyrosine aminotransferase, serine dehydrogenase, and phosphoenolpyruvate carboxykinase [32]. NF-kB and AP-1are involved in the regulation of genes related to the synthesis of adhesion molecules and cytokines. The inhibition of NF-kB by the corticosteroid–GR complex leads to a reduction in the production of interleukins such as IL-1, IL-6, interferon (IFN), and nitric oxide [33]. This effect takes more time to manifest but also leads to anti-inflammatory and immunomodulatory actions. It occurs through interactions with antigen-presenting dendritic cells, the modulation of IL-1 transcription, a decrease in the number of circulating CD4+ T-cells, and the inhibition of IL-1-dependent lymphocyte activation [18]. Higher doses of corticosteroids, on the other hand, act directly on the membrane (non-genomic effect), resulting in a faster response to the steroid. This is primarily due to three mechanisms: the release of proteins from the multiprotein complex after binding to the glucocorticoid receptor in the cytosol, interactions with membrane-bound receptors, and non-specific effects on the modification of the cell membrane induced by the steroids [34,35]. Higher doses of corticosteroids lead to the saturation of the receptors responsible for the long-term side effects.

### 4.3. Antimetabolites Drugs

There are three antimetabolite drugs available: AZA, MMF, and the enteric-coated formulation of mycophenolate sodium (EC-MPS). Only AZA and MMF are approved for LT [15]. Since the introduction of MMF, the use of AZA has declined, although evidence of a significant benefit of MMF over AZA remains limited [36,37,38].

AZA, synthesized in 1957, is a prodrug of 6-mercaptopurine, absorbed in the gastrointestinal tract and converted non-enzymatically into 6-mercaptopurine by intestinal, red blood, and hepatic cells [39,40]. In the liver, it forms thioinosinic acid, a purine analogue that interferes with DNA and RNA synthesis, exerting cytotoxic effects on leukocytes [40]. Enzymes such as hypoxanthine-guanine phosphoribosyltransferase (HGPRT) and other enzymes determine the formation of cytotoxic compounds, while xanthine oxidase and thiopurine S-methyltransferase (TPMT) create inactive compounds. The balance of these enzymes influences AZA’s efficacy and toxicity, with high TPMT activity reducing efficacy and low activity increasing toxicity. 6-Mercaptopurine inhibits leukocyte proliferation by disrupting nucleic acid synthesis and induces apoptosis through alternative pathways, including interactions with RAC-1, a GTPase that modulates T-cell migration and adhesion [41,42,43]. Additionally, 6-thioguanine metabolites downregulate anti-apoptotic molecules like BCL-XL, promoting lymphocyte apoptosis [44].

Mycophenolic acid (MPA) was identified in 1913 following the isolation of *Penicillium stoloniferum*, initially noted for its antibiotic, antiviral, and anti-inflammatory activities [45]. Although the compound was initially used for certain autoinflammatory diseases, its gastrointestinal side effects and association with the development of neoplasms led to its near abandonment [46]. Between the 1980s and 1990s, an ester of MPA, MMF, was developed, which demonstrated better bioavailability and fewer gastrointestinal side effects. This led the Food and Drug Administration (FDA) to approve it as a drug for the prevention of transplant rejection [47]. MMF is a prodrug of MPA that is almost completely absorbed in the small intestine and, in the bloodstream, is deesterified to MPA [48]. Only a small fraction of MPA enters the cell; the metabolite is subsequently converted into phenolic glucuronide and excreted via the urinary route [48]. Mycophenolate sodium, unlike MMF, has a slower absorption rate, resulting in a longer plasma concentration duration and a delayed time to reach peak serum concentration compared to MMF [48]. Like AZA, MMF acts on nucleotides by inhibiting guanine formation, specifically targeting the enzyme inosine monophosphate dehydrogenase (IMPDH), thereby effectively blocking the de novo synthesis of guanine [49]. Unlike other cells in the body, lymphocytes rely primarily on this pathway for guanine synthesis. The inhibition caused by MPA results in reduced lymphocyte proliferation and, consequently, a diminished antibody response, without inducing myelotoxicity [49]. MPA also appears to influence the expression of microRNAs in CD4+ T lymphocytes, reducing the expression of CD40 ligand. Additionally, it exerts effects on fibroblasts by inhibiting their proliferation [50,51,52,53]. They are less efficient than CNIs and are often used in combination with lower doses of CNIs to allow for dose reduction or the eventual discontinuation of CNIs [54].

### 4.4. Inhibitors of the Mammalian Target of Rapamycin (mTOR-I)

The mammalian target of rapamycin (mTOR) is a serine/threonine kinase that regulates a wide range of processes in eukaryotic cells in response to nutrients and growth factors [55]. mTOR is composed by two distinct complexes, mTOR complex 1 (mTORC1) and mTOR complex 2 (mTORC2). mTORC1 promotes cellular anabolism and suppresses catabolism, leading to the increased synthesis of proteins, lipids, and nucleotides, while reducing autophagy. In contrast, mTORC2 is involved in cell survival, cytoskeletal organization, gluconeogenesis, and lipogenesis [55,56]. In the context of LT, sirolimus (SRL) and everolimus (EVR) are used as mTOR inhibitors (mTOR-I). SRL was initially isolated from the fungus Streptomyces hygroscopicus and became the first mTOR-I to receive approval. EVR, which shares a similar structure with SRL, exhibits improved oral bioavailability and was approved by the European Medicines Agency (EMA) in 2003 as an immunosuppressive agent [56]. Other mTOR-I drugs, such as temsirolimus, deforolimus, ridaforolimus, and the novel class of mTOR-I known as TORKinibs, are not used in the context of LT and, therefore, will not be discussed in this article. SRL and EVR primarily inhibit mTOR within the mTORC1 complex by blocking substrate recruitment and subsequently interfering with their sites of action [57]. It has also been demonstrated that prolonged treatment with mTOR-I affects mTORC2 [58]. The effects of mTOR-I are multifaceted, as mTOR is involved in various processes, including protein synthesis, lipid metabolism, energy metabolism, autophagy, angiogenesis, epithelial-to-mesenchymal remodeling, and the development and function of the immune system. Regarding immunosuppression, mTOR-I can affect different cell populations, such as T and B lymphocytes and dendritic cells. mTOR-I are capable of reducing the functionality of T lymphocytes and regulating the synthesis of molecules like SP-1, CD62L, and CCR7, which reduce the ability of T lymphocytes to migrate out of lymphoid tissues [59,60]. They can also reduce the differentiation of CD4+ lymphocytes into Th1 and Th17 subsets without affecting the Th2 population as well as influence the differentiation of Treg cells, inducing apoptosis in activated CD4+ T-cells [61,62,63,64,65,66]. Dendritic cells (DCs) are antigen-presenting cells (APCs) with a critical role in immune response. The presence of mTOR-I can inhibit the maturation of DCs and induce apoptosis in monocyte-derived (mo)-DCs and CD34+-derived DCs but not in other cell lines such as macrophages and myeloid cells [67,68,69,70,71,72]. Mo-DCs in the presence of mTOR-I exhibit a reduced expression of antigen uptake receptors and decreased production of cytokines [70,73]. Additionally, mTOR-I can regulate plasmacytoid dendritic cells (pDCs), reducing the secretion of interferon α/β and impairing their ability to stimulate CD4+ T-cell activation. Regarding B lymphocytes, although further studies are needed for a more comprehensive understanding of this cell population, in vitro studies have demonstrated that mTOR-I can influence the proliferation and maturation of B-cells as well as reduce class switching recombination [60,74,75,76].

### 4.5. Basiliximab

One of the objectives of induction therapy is to inhibit T lymphocyte activation or block specific receptors (such as CD3 or IL-2R) through the use of monoclonal antibodies. Interleukin-2 (IL-2) is a cytokine responsible for the growth and activation of T and B lymphocytes as well as the activation of natural killer cells, macrophages, and monocytes [77]. The IL-2R is composed of three subunits: α-(CD25), β-(CD122), and γ-(CD132). The α-subunit is specific to IL-2R, and the binding between the α- and β-subunits is crucial for the activation of the IL-2R signaling pathway, which subsequently leads to the proliferation and clonal expansion of antigen-specific T and B lymphocytes involved in rejection [78]. The activation of these lymphocytes leads to further production of IL-2 and the activation of additional lymphocytes, which contribute to the destruction of the transplant’s vasculature [79]. Basiliximab is a chimeric monoclonal antibody, where the murine variable region is fused to a human immunoglobulin, and it inhibits T-cell proliferation by binding to IL-2R on activated T lymphocytes. It is used in induction therapy as a steroid- or CNI-sparing agent and in the treatment of steroid-resistant rejection [18]. The drug is administered in two doses: 20 mg within 2 h prior to LT and another 20 mg on the fourth day post-LT [80].

## 5. Side Effects

**Figure 2:**
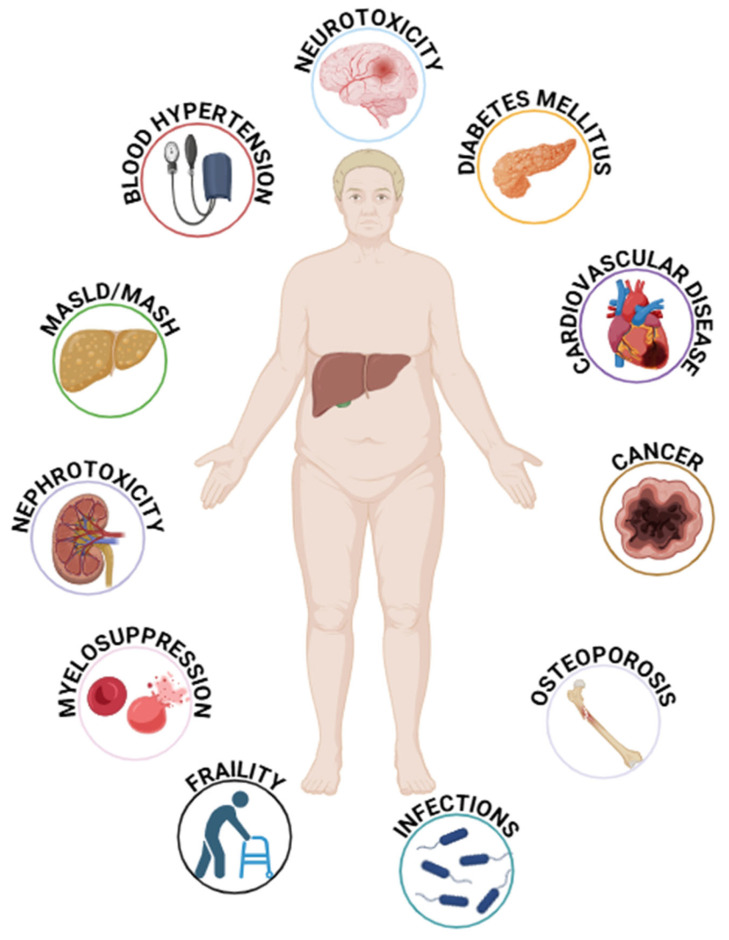
The main factors associated with immunosuppressive therapy in liver transplant patients. MASLD = Metabolic Associated Steatotic Liver Disease; MASH = Metabolic Associated Steatohepatitis.

Immunosuppression reduces immune surveillance against malignancies and infections and may have class-specific side effects. For instance, renal dysfunction is common with CNIs, while corticosteroids exacerbate metabolic profiles. Immunosuppression-related malignancy (16.4%) and opportunistic infections (10.5%) are the leading causes of death in long-term survivors after LT, followed by metabolic disorders, CV events, and renal dysfunction [18]. Moreover, a crucial aspect to consider is the significant pharmacological interactions both among immunosuppressive agents themselves and between immunosuppressants and other drugs. In vitro studies have demonstrated that CsA can increase SRL serum concentrations to a greater extent than TAC [81]. Drugs capable of inducing or inhibiting CYP3A4 or P-glycoprotein can alter the serum levels of CNIs and mTOR-I [82]. A review conducted by Van Matre et al. highlighted significant interactions between azathioprine (AZA) and ribavirin, mycophenolate mofetil (MMF) and amoxicillin/clavulanate, ciprofloxacin, and isavuconazole as well as between corticosteroids and ketoconazole, rifampin, isoniazid, erythromycin, ritonavir, and cobicistat [83]. This section discusses the long-term side effects of immunosuppressive therapies used in LT. Figure 2 highlights the potential side effects of immunosuppressive therapy in LT recipients.

### 5.1. Malignancy

LT recipients have an approximately 11-fold increased risk of developing malignancies compared to the general population [84]. The prevalence of de novo tumors specifically ranges from 3.1% to 14.4% at 5 years and from 10% to 14.6% at 10 years post-LT [85]. Malignancy remains the leading cause of mortality in this population [86]. Post-transplant neoplasms include both solid tumors and hematologic malignancies, which occur more frequently in LT recipients and often present with more aggressive behavior and earlier onset compared to the general population [87].

Skin cancer is the most common de novo malignancy, with squamous cell carcinoma and basal cell carcinoma being the most frequently observed types [86]. The overall incidence of non-melanoma skin cancer ranges between 16% and 22.5%, and it can occur at any time after LT, although it generally does not significantly impact mortality [88]. Established risk factors for skin cancer include advanced age, a history of extensive ultraviolet exposure, a tendency to sunburn easily, infection with human papillomavirus, a history of actinic keratosis, CD4 lymphocytopenia, blue or hazel eyes, and a higher intensity or prolonged duration of immunosuppressive therapy [87,88]. Notably, immunosuppression with CsA has been identified as one of the strongest independent predictors of skin cancer development. This association is likely attributable to CsA’s direct carcinogenic potential via activation of the Ras pathway, the promotion of tumor growth, increased metastatic potential through TGF-β1 activation, and the disruption of angiogenesis and apoptosis [86,89].

Post-LT lymphoproliferative disorder (PTLD) refers to a heterogeneous group of conditions characterized by abnormal lymphoid cell proliferation in immunocompromised individuals following solid organ transplantation [88]. PTLD exhibits a broad clinical spectrum, ranging from infectious mononucleosis with reactive polyclonal hyperplasia to systemic high-grade monoclonal lymphoma involving both nodal and extranodal sites. The latter manifests with greater aggressiveness and poorer clinical outcomes compared to lymphomas in non-transplanted populations [88]. The prevalence of PTLD in adult LT recipients is estimated to be 2–4%, making it the second most commonly diagnosed malignancy in this population [86]. EBV plays a pivotal etiological role in most PTLD cases, as immunosuppression compromises the activity of EBV-specific cytotoxic T lymphocytes, thereby permitting uncontrolled proliferation of EBV-infected B- or T-cells [88]. Management of PTLD typically begins with a 50% reduction in the dose of immunosuppressive therapy, which is often sufficient to induce remission while maintaining protection against graft rejection [88,90]. Among immunosuppressive agents, registry studies have identified a strong association between PTLD development and the use of CNIs and polyclonal T-cell-depleting antibodies, particularly ATG [90]. CNIs have been shown to facilitate PTLD by promoting EBV replication and enhancing the expression of EBV growth factors such as IL-1, IL-6, and transforming growth factor-β (TGF-β). Additionally, CNIs augment the expression of anti-apoptotic genes, which contributes to immune evasion and the persistence of infected cells [91]. The incidence of non-skin solid tumors in LT recipients exhibits significant variability, as most epidemiological data are derived from registry databases or single-center retrospective studies [86]. Although less frequent than skin malignancies, certain solid tumors are associated with extremely high mortality rates, for instance, 100% for lung cancer, 62.5% for esophageal and gastric cancers, 57% for head and neck cancers, and 50% for Kaposi’s sarcoma [87]. As in the general population, tobacco and alcohol consumption are well-established risk factors for oral, pharyngeal, laryngeal, esophageal, and upper airway cancers. These substances have a synergistic effect, increasing the risk of such malignancies by more than seven-fold in individuals who are both heavy drinkers and smokers [89]. Lung cancer, in particular, occurs at a two- to three-fold higher incidence in LT recipients compared to the general population and typically manifests 2–6 years post-transplantation, highlighting the critical need for aggressive smoking cessation strategies [86,88]. The incidence of colorectal cancer also appears to be elevated in the LT population. However, this increased risk is largely attributable to patients transplanted for primary sclerosing cholangitis, often in association with chronic inflammatory bowel disease [87,89]. Kaposi’s sarcoma, though rare in the general population, occurs more frequently in regions where human herpesvirus 8 (HHV-8) is endemic [89]. In individuals infected with HHV-8, the most significant risk factor for developing Kaposi’s sarcoma is the degree of immunosuppression. Management typically involves tapering immunosuppressive therapy, with or without the addition of chemotherapeutic agents [87].

To date, no robust evidence has confirmed an increased post-transplant risk for genitourinary cancers, including bladder and renal cancers, or for non-breast gynecological cancers, such as cervical and ovarian cancer, in LT recipients [87,89]. Nevertheless, the development of these malignancies has been associated with well-established environmental risk factors, such as smoking for bladder cancer and infection with oncogenic HPV types for cervical cancer [89]. In contrast, prostate and breast cancer, which rank among the most common malignancies in the general adult population, do not appear to occur at an increased frequency in LT recipients compared to age-matched controls [87,89].

Cumulative exposure to TAC levels exceeding 10 ng/mL within the first month and 8 ng/mL within the first three months post-LT has been identified as a predictor of immunosuppression-related post-transplant de novo malignancies, particularly skin cancer, colorectal cancer, lung cancer, and HCC recurrence [92].

Notably, the use of SRL or EVR has been associated with a reduced risk of both de novo and recurrent post-transplant malignancies, including HCC, due to their antiproliferative properties [18]. A recent retrospective study conducted by De Simone et al. showed that LT patients with HCC receiving EVR had a reduced risk of HCC recurrence compared to those on TAC [93].

### 5.2. Infections

Infections remain the leading cause of mortality and morbidity among LT recipients, with reported rates reaching up to 80% [94]. Bacterial infections account for approximately 70% of all infections following LT, followed by fungal and viral infections [95]. These infections generally follow a distinct temporal pattern: during the first month post-transplant, nosocomial infections associated with surgical procedures and invasive post-operative interventions (e.g., mechanical ventilation, indwelling vascular, and urinary catheters) predominate. Between 2- and 6-months post-transplant, when immunosuppression reaches its peak, opportunistic infections and the reactivation of latent infections represent the primary sources of morbidity. Beyond 6 months, the frequency of infections declines, with community-acquired infections becoming the principal concern [15,94].

During the first month after transplantation, bacterial infections are most prevalent and typically involve the surgical site, abdomen, bloodstream, and the urinary and respiratory tracts [94]. Common causative pathogens include *Enterococcus* spp., *Streptococcus viridans*, *Staphylococcus aureus*, and members of the *Enterobacteriaceae* family [94]. Alarmingly, multidrug-resistant (MDR) infections are on the rise, with a reported prevalence of 18–50%, posing a significant challenge in clinical management. Particularly concerning are infections caused by *Methicillin-Resistant Staphylococcus aureus* (MRSA), *Vancomycin-Resistant Enterococcus* (VRE), *carbapenem-resistant Enterobacteriales*, and extended-spectrum beta-lactamase (ESBL)-producing *Pseudomonas aeruginosa* and *Acinetobacter* species [94].

During the period from 1 to 6 months post-LT, recipients are at risk of developing opportunistic infections such as candidiasis, aspergillosis, and toxoplasmosis [96]. Invasive fungal infections are reported in 5% to 42% of LT recipients [96]. Among these, candidiasis (60–80%) and aspergillosis (1–8%) are the most frequently observed mycoses, with high mortality rates of 30–50% and 65–90%, respectively [96,97].

Several risk factors predispose recipients to invasive fungal infections, including the preoperative use of broad-spectrum antibiotics, a pretransplant diagnosis of fulminant hepatic failure, concurrent CMV or HCV infection, serum creatinine levels > 3 mg/dL, prolonged intensive care unit stays, and extended surgical durations exceeding 11 h [96].

Toxoplasmosis, although less common in LT recipients, has an incidence rate ranging from 0.9% to 2.5%. Despite its rarity, toxoplasmosis carries a high morbidity and mortality rate, likely attributable to delays in diagnosis [98].

Infections caused by *Pneumocystis jirovecii* and herpesviruses (CMV, EBV, HSV, and VZV) have become less frequent in LT recipients due to the routine use of prophylactic therapy [96]. Recent studies indicate that the overall incidence of *P. jirovecii* infections in LT recipients has decreased significantly, from as high as 10% in the absence of prophylaxis [99] to less than 2% in patients receiving prophylaxis [94]. The risk of *P. jirovecii* pneumonia in LT recipients remains closely linked to the intense immunosuppressive regimens used to manage acute cellular rejection. Other risk factors include prolonged courses of high-dose corticosteroid therapy, CMV infection, persistent neutropenia, and exacerbations of autoimmune diseases [99].

Regarding HBV, it has been known since the 1980s that viremic patients undergoing transplantation while in a viremic phase are at the highest risk of HBV recurrence. Therefore, all patients on the LT waiting list should be treated with nucleos(t)ide analogues (NA) with the goal of achieving undetectable viral concentrations in plasma [15]. The combination of hepatitis B immunoglobulin (HBIG) and a potent NA is recommended post-transplant to prevent HBV recurrence. Low-risk patients for recurrence may also discontinue HBIG [100]. In patients with latent HBV infection, lifelong prophylaxis with lamivudine is required [100].

It is important to note that in immunosuppressed patients, other viruses can become chronic. One example is the hepatitis E virus (HEV), which accounts for approximately 20 million infections annually and is not routinely tested by organizations involved in blood collection and storage [101,102]. Moreover, the virus can be acquired through the consumption of inadequately cooked pork or wild boar meat. The first-line treatment for patients with chronic HEV involves reducing immunosuppressive therapy, which may lead to viral clearance through the host’s immune system. In other cases, the use of ribavirin, sofosbuvir, or pegylated interferon-alpha may be required [102,103].

CMV infection typically manifests within the first three months following LT and is the most common viral complication, accounting for 54.3% of all viral infections in this population [104]. CMV has been shown to quadruple the risk of acute and chronic allograft rejection by potentiating alloantigens and impairing CD4 T-cell and macrophage functions [104,105]. These indirect effects further predispose patients to invasive fungal infections, bacteremia, EBV-associated PTLD, and CV complications [104,105]. CMV viremia should be monitored at least during the first month post-LT [15]. Proper CMV prophylaxis must be implemented to reduce the incidence of infection [15]. The treatment of choice is intravenous ganciclovir or oral valganciclovir and should be considered for all patients with persistent CMV viremia and those who develop infection-related symptoms [15].

The incidence of VZV infection after LT is reported as 17.83 per 1000 person-years, a rate significantly higher than that in the general population [106]. Older age and exposure to MMF or AZA have been identified as independent risk factors for VZV infections in this context [106,107].

*Cryptosporidium* spp. infection is caused by protozoa that contaminate food or water and typically induce diarrhea in immunocompetent hosts [108]. In immunosuppressed patients, more severe conditions, such as disseminated disease, may occur [109]. Cryptosporidiosis in immunosuppressed patients can also result in fatal forms [110]. The first-line therapy in these cases is the reduction of immunosuppression. If this is not possible or sufficient, nitazoxanide may be administered alone or in combination with azithromycin [110,111].

Active tuberculosis (TB) in LT recipients typically arises from the reactivation of latent Mycobacterium tuberculosis infection, with most cases occurring within the first year post-LT [96,112]. The prevalence of M. tuberculosis infection in LT recipients varies significantly by geographic region, reported as 0.6% in the United States, 1.4% in European Union countries, and 2.2% in non-US/non-EU countries. Mortality associated with post-LT TB ranges from 18% to 36% [94]. Pulmonary TB is the most common clinical manifestation, accounting for up to 60% of cases in this population [94]. However, extrapulmonary and disseminated TB presentations are significantly more frequent in transplant recipients compared to immunocompetent individuals [112].

TB prophylaxis in liver transplant patients is a critical consideration due to their immunosuppressed condition. Additionally, in organ transplant recipients, the likelihood of developing active TB is between 20 and 74 times higher than in the general population, with the majority of cases resulting from the reactivation of latent TB [113,114]. Screening for latent TB is important both due to the high incidence of the disease and the associated risk of mortality from active TB in solid organ transplant recipients [115]. There are no specific indications from the EASL guidelines regarding the treatment of latent TB; however, the WHO guidelines classify transplant recipients as high-risk individuals for TB and recommend treatment for them [15,116]. The drugs typically used in these contexts are isoniazid and rifampin. Close laboratory monitoring should be conducted in these patients, as these medications can increase transaminase levels and cause interactions with immunosuppressive drugs [117].

EVR has been linked to an increased risk of interstitial pneumonia, which is dose-dependent and resolves upon the discontinuation of EVR; however, it has not been associated with an increased risk of opportunistic infections [18]. Finally, corticosteroids have been identified as a risk factor for invasive aspergillosis [97].

### 5.3. Metabolic Disorders

MetS, characterized by obesity, dyslipidemia, hypertension, and hyperglycemia, represents one of the most common complications following LT, with reported prevalence rates ranging from 44% to 58% across various studies [118]. Alongside immunosuppression, MetS is recognized as a key risk factor for the development of CV disease in LT recipients, which accounts for 19% to 42% of all non-graft-related deaths in this population [118].

The prevalence and severity of obesity notably increase post-LT, rising from 23.8% at four months to 40.8% by three years after transplantation [119]. Charlton and colleagues observed that patients with obesity prior to transplantation tend to remain obese, while overweight individuals often progress to obesity following the procedure [119]. In their study, individuals with normal weight at baseline who received EVR combined with reduced TAC exhibited less weight gain compared to those treated with TAC alone [119].

This finding aligns with evidence suggesting that mTOR-I, such as EVR, may alter fatty acid uptake and triglyceride synthesis pathways, in addition to reducing adipocyte cell proliferation, thereby contributing to weight modulation [120].

Blood hypertension is a common complication after LT, affecting up to 70% of long-term patients. This condition is attributed not only to post-LT hemodynamic changes but also to immunosuppressive therapy [121]. In a study evaluating the effects of CNIs and EVR two years post-transplantation, blood hypertension was observed in 57.6% of patients treated with TAC, 50.0% of those receiving EVR, and all patients on CsA [122].

Several pathogenic mechanisms underlie CNI-related hypertension, with key contributors including renal vasoconstriction, reduced glomerular filtration rate, and enhanced sodium absorption in the proximal renal tubule. Additionally, CsA increases sympathetic nervous system activity through endothelial dysfunction, further elevating blood pressure [122].

While studies in kidney transplant recipients suggest that mTOR-I may exacerbate hypertension when combined with CNIs [107], research on LT recipients has produced inconsistent findings [123,124,125]. Furthermore, corticosteroids contribute to blood hypertension via their mineralocorticoid effects, increased vascular resistance, and enhanced cardiac contractility [118].

Immunosuppression plays a significant role in the development of dyslipidemia following LT. Findings from three randomized trials evaluating early conversion from CNI therapy to either SRL or EVR revealed elevated levels of total cholesterol and low-density lipoprotein cholesterol (LDLc) in the mTOR-I group [126]. This is consistent with the lipogenic effects of mTOR-I, which inhibit lipid uptake into adipocytes and promote lipolysis [126]. CNIs have also been linked to hypercholesterolemia and hypertriglyceridemia, with CsA showing a higher prevalence of these complications (30% and 33%) compared to TAC (6% and 3%) [127]. CsA contributes to dyslipidemia by reducing biliary cholesterol excretion and inhibiting LDLc receptors, leading to increased cholesterol levels in the bloodstream [118].

Steroids induce lipogenesis by stimulating acetyl-CoA carboxylase activity and impairing LDLc receptor function. However, their impact on lipid metabolism is typically transient due to their limited long-term use in post-transplant regimens [120].

Post-transplant diabetes mellitus (PTDM) is a common metabolic complication that significantly compromises long-term survival by increasing the risk of end-stage renal disease, infection, graft failure, CV events, and mortality. Risk factors associated with the development of PTDM are high recipient age at the time of transplant, male gender, the indication for LT (cirrhosis due to MASH, HCV, or alcohol or cryptogenic cirrhosis), an increase in body mass index after transplantation, smoking, and immunosuppression [118,121]. LT recipients on SRL have the highest incidence of PTDM (27%) at 2–3 years after transplant compared to TAC and CsA (23% and 15%, respectively) [128].

While acute use of SRL has been shown to prevent insulin resistance, chronic use of SRL has been associated with the development of glucose intolerance and hyperlipidemia in animal models, particularly rats [129]. These effects are primarily attributed to a reduction in β-cell mass, decreased hepatic insulin clearance, and increased hepatic gluconeogenesis, all of which are induced by direct mTOR-I [129].

When comparing the two CNIs head-to-head, CsA was found to induce a higher incidence of PTDM one year after LT compared to TAC (18% vs. 11%, respectively). However, after two to three years, the incidence of PTDM was higher among patients treated with TAC compared to those on CsA (23% vs. 15%, respectively) [128]. Both TAC and CsA have similar inhibitory effects on pancreatic β cells, leading to reduced insulin synthesis and increased insulin resistance [130]. The difference between CsA and TAC may be attributed to the potency of calcineurin inhibition. The superior effect of TAC in preventing rejection has been found to be accompanied by an increased rate of diabetes [131]. Additionally, corticosteroids induce insulin resistance in a dose-dependent manner by decreasing β-cell insulin production, enhancing gluconeogenesis, and reducing peripheral glucose utilization. However, corticosteroids are typically not used over the long term, so their impact on the MetS is generally transient [120].

MASLD and MASH are well-established metabolic complications that can affect patients after LT. In a meta-analysis, the mean 1-, 3-, and 5-year incidence rates of recurrent and de novo MASLD were 59%, 57%, and 82% and 67%, 40%, and 78%, respectively [121]. In contrast, the incidence of post-transplant MASH was significantly higher for recurrent MASH (53%, 57.4%, and 38% at 1, 3, and 5 years after LT, respectively) compared to de novo MASH (13%, 16%, and 17%, respectively) [121]. Cirrhosis was reported in patients with recurrent or de novo MASLD at a rate of 11–14% and 1%, respectively, at 5 years post-LT [121]. Despite MASLD being an extremely common post-transplant complication, no significant difference in graft survival between patients with recurrent MASH or MASLD has been observed [132]. However, it is important to note that although long-term survival rates are not significantly different, these patients remain at an increased risk for CV events due to underlying metabolic disease.

### 5.4. Nephrotoxicity

Renal dysfunction following LT can result from perioperative acute kidney injury (AKI), pre-existing chronic kidney disease (CKD), or the development of CKD after the transplant. AKI after LT is a common complication, with a reported incidence ranging from 30% to 60%, and is caused by acute tubular necrosis in most cases [133]. The incidence of CKD has been reported to range from 4.0% to 27.5% within one year and 30% to 50% within 10 years post-surgery [133]. Independent risk factors for new-onset CKD include higher intraoperative blood loss, blood transfusions leading to renal ischemia-reperfusion injury, postoperative AKI, postoperative hypertension, and elevated average plasma concentrations of CNIs [134].

CNIs are responsible for over 70% of cases of end-stage renal disease (ESRD) after LT [15]. CNIs cause a dose-dependent afferent renal arteriolar vasoconstriction, which is typically reversible. However, prolonged ischemic glomerular and tubular injuries can lead to irreversible tubulointerstitial fibrosis [18]. Additionally, the side effects of CNIs, including blood hypertension and diabetes, further exacerbate renal damage [134]. It has been demonstrated that conversion from CNIs to mTOR-I improves renal function in patients with plasma creatinine levels > 1.5 mg/dL and stabilizes proteinuria and creatinine levels in those with diabetic and hypertensive nephropathy after LT [135]. Specifically, the use of EVR combined with CNI minimization, resulted in a significant improvement in renal function at one year, with an estimated glomerular filtration rate (GFR) increase of 10.2 mL/min (95% CI: 2.75–17.8) in a meta-analysis [136]. Furthermore, the combination of MMF and SRL showed a significantly greater improvement in renal function compared to MMF and CNIs in a randomized trial with a median follow-up of 1.4 years, with a mean percentage change in the GFR of 19.7  ±  40.6 versus 1.2  ±  39.9, respectively (*p*  =  0.0012) [137].

Immunosuppressive regimens using MMF or Basilixumab have been shown to offer a better renal profile compared to conventional CNI-based regimens. However, it is challenging to accurately assess the real impact of both MMF and Basilixumab, as the data supporting their use come from small patient cohorts [138,139,140,141,142].

### 5.5. Neurotoxicity

The overall prevalence of neurological symptoms or disorders after LT is reported to be around 25%, which is notably higher than that seen in other organ transplants such as the kidneys (0.5%) and heart (3.6%) [143]. Neurological complications are most common during the early period (1–3 months) following LT and can range from mild to severe. Mild complications include tremors, headache, sleep disorders, and peripheral neuropathy, which often resolve spontaneously. Severe complications, such as seizures, stroke, posterior leukoencephalopathy, central pontine myelinolysis syndrome, toxic-metabolic encephalopathy, and central nervous system (CNS) infections, can have significant and lasting consequences [143,144].

Among the immunosuppressive drugs used after LT, MMF is unique in not being associated with neurotoxic side effects [143,144]. In contrast, CNIs such as CsA and TAC are commonly linked to neurotoxicity. The manifestations of CNI-related neurotoxicity can range from tremors and headache to more severe symptoms, such as seizures, altered mental status, stupor, coma, confusion, agitation, and psychosis [145]. Approximately 18% of moderate-to-severe neurological complications occurring within 4 weeks after LT are related to CNI use. Risk factors for the development of CNI-related neurotoxicity include older recipient age, a history of pre-LT hepatic encephalopathy, pre-LT hyponatremia, surgical time longer than 7 h, and post-LT hyponatremia [145]. Neurotoxicity is observed in about 10% to 28% of patients treated with CsA and 21% to 32% of those treated with TAC [146].

The exact neurotoxic mechanism of CNIs remains unclear. However, it is hypothesized that the neurotoxicity may be related to the high-affinity binding of CNIs to immunophilins, which reach elevated levels in central nervous system (CNS) cells [143]. Oligodendrocytes, which contain higher levels of calcineurin compared to astrocytes, may be particularly vulnerable to this binding, explaining the selective toxic effects on these cells and why CsA exposure can lead to demyelination [143]. Both TAC and CsA are known to reduce endothelial nitric oxide production, which can lead to severe vasoconstriction in cerebral vessels and resulting ischemia [143]. Additionally, the reduction in calcineurin phosphorylation caused by CNIs disrupts GABAergic neurotransmission, which can predispose patients to seizures, while altering serotonergic neurotransmission may facilitate tremors [143].

In contrast, mTOR-I binds to a different site on immunophilins and does not cause neurotoxic effects. Studies evaluating the conversion to SRL or EVR following CNI-related neurotoxicity in LT patients have shown that neurological complications improve or resolve in 80–100% of cases [146,147,148].

Corticosteroids are associated with neuropsychiatric side effects in 3–4% of LT patients. The most common side effects include mood changes, behavioral disorders (including psychotic reactions), and neuromyopathy, which manifests as muscular weakness [143,144].

### 5.6. Myelosuppression

Immunosuppressive medications can contribute to the development of anemia following LT due to their bone marrow-suppressive effects. Controlled trials comparing different immunosuppressive regimens have reported an incidence ranging from 1% to 53% [149]. In the European FK506 Liver Study comparing CsA and TAC, the incidence of anemia was 1% in the CsA group and 5% in the TAC group [149]. In contrast, the US FK506 Liver Study group reported significantly higher anemia incidences, with 38% of patients in the CsA group and 47% in the TAC group [149]. Anemia in this context is typically mild to moderate (mean hemoglobin 10.3 ± 1.6 g/dL), and in most cases, the discontinuation of CNIs leads to complete hemoglobin recovery within three weeks [150]. Additionally, sporadic cases of thrombotic thrombocytopenic purpura and red cell aplasia have been reported with TAC use [150].

The use of mTOR-I has also been associated with anemia, likely due to iron deficiency secondary to an inflammatory state [151]. The myelosuppressive effects of mTOR-I result from the inhibition of signal transduction pathways shared by several cytokine receptors, including those that stimulate platelet, leukocyte, and erythrocyte production [151]. MMF is generally well tolerated; however, its most common side effects include leukopenia, anemia, and thrombocytopenia [152]. A randomized study comparing MMF and AZA, both in combination with CsA and corticosteroids in LT recipients, reported anemia as an adverse event in 43% of patients in the MMF group and 53% in the AZA group [36]. The incidence of anemia with MMF is dose-dependent and can generally be managed by reducing the MMF dose. Treatment discontinuation is rarely required [152].

### 5.7. Osteoporosis

Patients with liver cirrhosis exhibit a higher prevalence of metabolic bone disease compared with the general population, with an estimated prevalence ranging from 12% to 55% [153,154]. This condition is associated with a higher propensity for fragility fractures, with an incidence rate of approximately 40% [155,156]. Several factors contribute to the pathogenesis of osteoporosis in transplanted patients: pre-existing conditions prior to transplantation, factors such as the use of immunosuppressive drugs and lifestyle choices, and imbalances in the parathyroid–calcium–vitamin D and pituitary–gonadal axes [157,158].

Furthermore, patients on the transplant waiting list often experience complications associated with organ failure, such as cachexia, nausea, inadequate nutrition, reduced mobility, and hypogonadism, which are particularly common in end-stage liver disease patients [158]. Additionally, certain medications commonly used in liver disease, such as corticosteroids, loop diuretics, ribavirin, and tenofovir, may negatively affect bone balance [158].

In a recent study by Zavatta et al. [159], a 42% prevalence of osteoporotic fractures was identified among LT recipients, most commonly affecting the thoracic vertebrae, femur, humerus, and ribs. An interesting finding from the study is that a 1-point reduction in BMI was associated with a 5.8% increase in the risk of fragility fractures (*p* < 0.05) [159].

After transplantation, bone mineral density decreases rapidly within the first 3–6 months but tends to recover by two years post-LT [160,161,162].

CNIs have been associated with bone mineral density loss following LT [160], with bone density being inversely correlated with serum CsA levels at one-year post-transplant [163].

Corticosteroids also induce significant bone mineral loss, particularly in the short term. However, steroid therapy in LT patients should generally be limited to the early post-transplant phase or episodes of liver rejection [15,154,158,164]. A proper supplementation of calcium and vitamin D should generally be ensured for patients with deficiencies, and the use of bisphosphonates may be considered for patients at high fracture risk. Denosumab has been shown to significantly reduce osteoporosis in transplanted patients, as demonstrated in a retrospective study [165].

According to a study by Bueno et al., LT recipients appear to have a higher propensity for developing osteonecrosis of the jaw when treated with bisphosphonates compared to those not receiving bisphosphonates [166].

### 5.8. Frailty

Frailty is a multidimensional concept primarily described in geriatrics. It is defined as a syndrome characterized by weight loss, exhaustion, weakness, reduced gait speed, and decreased physical activity [167]. Frail patients are at a higher risk of accidental falls, loss of independence, reduced quality of life, increased hospitalization rates, and greater mortality [167]. There is limited but growing evidence describing the post-LT progression of frailty. Generally, a worsening of frailty is observed at three months post-LT, followed by an improvement at twelve months [168]. The mechanisms by which individual immunosuppressants contribute to sarcopenia and, more broadly, frailty in post-LT patients remain unclear. However, the deterioration of metabolic, renal, and neurological or psychological profiles may exacerbate frailty in these patients. Additionally, inadequate lifestyle factors and limited social support networks may further contribute to this condition [169,170].

## 6. Is Immunosuppressive Withdrawal Feasible After LT?

As mentioned earlier, among the various solid organ transplants, the liver is the organ with the best immunotolerance, requiring comparatively lower levels of immunosuppression than other organs [15,16]. The 2024 EASL guidelines for LT recommend a gradual reduction of immunosuppression under expert supervision, emphasizing that long-term survivors are often overly immunosuppressed [15]. Several studies have explored the possibility of discontinuing immunosuppression in LT patients [171,172,173,174,175,176,177,178,179,180,181,182]. Although the EASL guidelines do not recommend the suspension of immunosuppression either as a standard of care or in patients with an extended postoperative course without transplant rejection [15], some estimates suggest that 20–40% of LT patients may maintain immunotolerance after discontinuation of immunosuppression [183]. Additionally, beyond the spontaneous operational immunotolerance observed in these patients, a question currently being explored is the potential contribution of pharmacologically induced immunotolerance, or therapeutic operational immunotolerance [184]. Therefore, in addition to the search for a greater understanding of the underlying mechanisms of immunosuppression, efforts are being made to identify biochemical or histological factors that could define patients at high risk of rejection. The decision to modulate or discontinue immunosuppression may stem from the side effects of immunosuppressive drugs and the risk of rejection, and this decision should be personalized for each patient. Currently, no tools are available to identify which patients are immunotolerant; liver transaminases may be normal despite increased hepatic fibrosis, as characterized histologically in cohorts of patients who have discontinued immunosuppression [185]. Some interesting lessons have been provided by Benitez et al. [186], Shaked et al. [187], and Duizendstra et al. [182]. In the study conducted by Benitez et al. on nearly 100 LT patients followed for 3 years with serial biopsies, it was observed that the likelihood of immunotolerance was associated with older age, longer time since transplantation, and male sex [186]. Specifically, the percentage of immunotolerant patients was 20% in total, with 78% in patients who had undergone LT at least 10.6 years earlier, which decreased to 38% if the transplant had been performed 5.7 years earlier. When the LT was performed less than 5.7 years prior, the percentage dropped to 0% [186]. In the study conducted by Shaked on 77 LT patients, a progressive reduction in immunosuppressive therapy was induced until withdrawal in patients who had undergone transplantation 1–2 years earlier, with biopsy verification at three months and after immunosuppressive withdrawal [187]. In this study, the percentage of immunotolerant patients was 19%, with better outcomes in terms of survival, secondary opportunistic infections, reduced renal function, and secondary malignancies [187]. Duizendstra et al. conducted a retrospective case-control study on the effects of complete immunosuppression withdrawal in patients with a median time since LT of 10.8 ± 5.1 years [182]. Patients who underwent immunosuppressive withdrawal showed improvements in LDL values, a reduction in new infections, and resolution of persistent infections; however, no improvement in renal function, reduction in new diagnoses of T2DM, hypertension, malignancies, or CV events was observed [182]. A smaller-scale study analyzed 15 non-immune, non-viremic LT recipients undergoing SRL therapy for at least three months and at least three years post LT [188]. Of these 15 patients, 8 were found to be immunotolerant one year after discontinuation of SRL therapy. Immunotolerant patients exhibited a higher percentage of HLA-DR+CD11c+ILT3+ILT4+ DCs prior to weaning (*p* < 0.01) [188]. Further studies are required to confirm these findings. In a recent multinational randomized controlled trial (RCT), a 28% discontinuation rate of immunosuppressive therapy without the development of allograft rejection was reported, with 16% of patients achieving operational immunotolerance at one year [189]. An interesting finding reported by Vionnet et al. is the overexpression of interferon-stimulated genes (ISG15, ISG20, OASL, OAS1, IFI6, IFITM1, IFI35) in immunotolerant patients [189].

The results of these studies raise several considerations regarding immunosuppression withdrawal, namely that only a small, selected portion of patients in these studies were found to be immunotolerant, and the long-term benefits remain uncertain. Moreover, the search for tools capable of predicting which patients may develop immunotolerance is crucial in identifying the small subset of patients who could benefit from a reduction or discontinuation of immunosuppressive therapy. Further studies will be needed to identify ideal markers and timing of immunosuppression withdrawal.

## 7. Unconventional Strategies in Post-Transplant Immunosuppression

Although the exact mechanisms by which the immune system interacts with the LT remain incompletely understood, the past 15 years have witnessed the discovery and development of novel immunosuppressive molecules that could improve long-term outcomes for LT patients.

### 7.1. Costimulatory Blockade Agents

Costimulation represents a crucial step in the activation of an antigen-specific immune response, and its inhibition could theoretically lead to clinical operational tolerance. The first study in the context of LT was conducted in the late 1990s using an anti-CD2 monoclonal antibody [190]; in this context, the authors concluded that the incidence of moderate-to-severe rejection was lower in the experimental group compared to the group receiving TAC [190].

Currently, there are two clinical studies involving Siplizumab, an anti-CD2 monoclonal antibody capable of inducing antibody-dependent cell-mediated cytotoxicity among NK cells [NCT06019507, NCT06455280].

Another drug capable of blocking costimulation is belatacept. This is a fusion protein composed of an FC fragment of a human immunoglobulin associated with an extracellular domain of the Cytotoxic T-Lymphocyte Antigen 4 (CTLA-4, also known as CD152) molecule, a receptor expressed on CD4+ and CD8+ T lymphocytes. It can bind to CD80 and CD86 on APCs, resulting in a significant reduction in the activity of effector lymphocytes and Tregs [191,192]. It is currently approved for kidney transplantation with the aim of reducing the dosage of CNIs during the maintenance phase [193]. In LT, the results are limited to a single phase 2 study, which showed disappointing outcomes. In fact, the graft survival at one year was lower compared to the group receiving TAC and MMF. The arm with more intensive belatacept therapy demonstrated higher mortality, often leading to early treatment discontinuation due to a greater number of graft losses [194]. Some case reports and case series on the use of belatacept in LT patients experiencing rejection or in patients with combined kidney and LT have reported the resolution of rejection in specific contexts or the reduction of CNI dosage with relatively few side effects [194,195].

The blockade of the interaction between CD40 and CD40L is another mechanism under investigation; notably, two monoclonal drugs, bleselumab and iscalimab, have already been evaluated in clinical research [192]. However, only iscalimab has been studied in LT patients in a phase 2 RCT. Nonetheless, the study was terminated due to the less favorable efficacy of iscalimab (CFZ533) in LT patients compared to TAC[NCT03781414].

### 7.2. T-Cell Exhaustion

Exhausted T lymphocytes are dysfunctional T-cells characterized by a progressive loss of cytokine production (interferon-γ and tumor necrosis factor (TNF)-α), an increased expression of inhibitory receptors, and reduced proliferative capacity. T-cell exhaustion can occur as a result of continuous and prolonged exposure to antigens [192]. At present, the mechanisms underlying T-cell exhaustion are not well understood. However, it has been observed that T-cell exhaustion is associated with improved graft function and that the only inhibitory receptor identified is Fcγ RIIb [196,197]. Fcγ RIIb appears to play a role in both innate immunity, by acting on CD8+ T lymphocytes, and in inflammation, by acting on B lymphocytes. Therefore, the development of an Fcγ RIIb agonist could represent a significant step forward in achieving operational clinical tolerance. T-cell exhaustion can be achieved through induction with depleting agents and the progressive reduction of CNIs [198].

The available techniques include the administration of depleting agents capable of upregulating various immune checkpoints [199]. Muromonab-OKT3, a murine-derived monoclonal antibody targeting CD3 on human T lymphocytes, was the first to be used in solid organ transplantation and successfully reversed graft rejection [200,201]. The administration of Muromonab-OKT3 induces T lymphocyte depletion through immune-mediated phagocytosis or via TCR internalization [202]. However, due to the potential for polyclonal expansion, the risk of cytokine storm development, drug sensitization, the increased incidence of de novo neoplasms, and the lack of documented long-term effects, the drug was withdrawn from the market [203]. Another depleting agent used in LT is Alemtuzumab, a monoclonal antibody targeting CD52, which allows for a reduction in acute rejection episodes while using lower doses of immunosuppressive medications [204,205]. However, an increase in complications due to viral reactivations has been observed, leading to its discontinuation [206,207,208].

Calcineurin and NFAT are important in the induction of T-cell exhaustion, as they are capable of increasing the transcription of Thymocyte selection-associated high mobility group box protein (TOX), which leads to T-cell exhaustion [209]. Since CNIs hinder this process, several studies have evaluated the use of antibodies against lymphocytes to minimize CNI doses. However, the results obtained have not yet been confirmed by RCTs [210,211,212].

### 7.3. Selective Inhibitors of T-Cell Signaling

The Janus tyrosine kinase (JAK)/signal transduction and activators of transcription (STAT) system is responsible for signal transduction mediated by various cytokines and growth factors [213]. Dysregulation and mutations of the JAK/STAT pathway are associated with the development and progression of various diseases, such as solid tumors, autoimmune diseases, and inflammatory disorders [214,215]. Indeed, JAK/STAT upregulation can lead to the activation and expansion of autoreactive T lymphocytes or APCs, playing a role in this system in the development of allograft rejection [216]. There are several JAK/STAT inhibitors, among which tyrphostin AG490, a JAK2 inhibitor, has been shown to inhibit T-cell proliferation and render them hyporesponsive when stimulated by alloantigens or by CD3 or CD28 [217]. Ruxolitinib has been approved by the FDA to improve steroid-refractory graft-versus-host disease in stem cell bone marrow transplantation [218], while Tofacitinib has shown improvements in kidney transplantation [219]. Regarding LT, there are no studies on JAK/STAT inhibitors in LT patients, but in vitro studies have shown a reduction in liver damage induced by the ischemia/reperfusion process in mice [220].

### 7.4. Monoclonal Antibodies

Monoclonal antibodies can be used in the immediate post-transplant period to prevent acute rejection by inhibiting a fulminant T-cell response before conventional immunosuppressive drugs reach adequate concentrations [221]. Furthermore, the use of these drugs may lead to a reduction in the dose of CNIs and could be employed to replace steroids in certain immunosuppressive protocols, according to some authors [222,223,224]. In addition to muromonab, previously discussed, antibodies against the interleukin-2 receptor (such as basiliximab and daclizimab) induce immunosuppression by reducing T-cell expansion and have been used as prophylaxis for acute organ rejection following transplantation [225,226,227]. A meta-analysis has shown that the use of interleukin-2 receptor inhibitors is associated with a lower risk of acute organ rejection but does not lead to an increase in survival [221].

## 8. Conclusions

Immunosuppression is a cornerstone of LT therapy. Although the liver is a more immunotolerant organ and requires a lower dosage of immunosuppressants, there are currently no criteria to guide its withdrawal under specific conditions. TAC represents the cornerstone of immunosuppressive therapy, with levels maintained at 6–10 ng/mL during the first month, followed by 4–8 ng/mL thereafter. The combination of TAC with other immunosuppressive drugs (MMF, AZA, or mTORi) is recommended to achieve lower TAC trough levels than those required for monotherapy, thereby helping to preserve renal function. The use of basiliximab for induction therapy (with a delayed introduction of TAC) is advisable in recipients at a high risk of renal dysfunction [15]. The adverse effects of immunosuppression, both general and molecule-specific, in addition to the changing population of transplant recipients—particularly with a significant contribution from MASH-related cirrhosis—have increased focus on the metabolic and CV aspects of LT patients, alongside the usual surveillance for rejection and neoplasms [1,2]. This highlights an even greater need for personalized immunosuppressive therapy, aiming to balance the complications of immunosuppression with the risk of liver rejection. A crucial future objective will be the development of new immunosuppressive strategies with a reduced metabolic impact. These approaches could lower the incidence of metabolic syndrome, post-transplant diabetes mellitus, and dyslipidemia, thereby improving patients’ quality of life and reducing the risk of CV complications. Simultaneously, the introduction of novel molecules or pharmacological combinations may enable more targeted management while minimizing class-specific side effects of currently used drugs.

Another strategic goal is the identification of biomarkers or clinical-laboratory tools capable of identifying immunotolerant patients, who can safely discontinue immunosuppressive therapy. This innovation would further reduce the risks associated with chronic immunosuppression, improve long-term outcomes, and pave the way for a more selective and safer approach to patient management. In this regard, encouraging results have been demonstrated by deep learning and machine learning models in artificial intelligence, both in the pre-LT and post-LT settings [228,229,230]. Machine learning algorithms can be used to predict the outcome of a new observation based on a training set containing previous observations where the outcome is known [231]. Regarding the pre-LT setting, some artificial intelligence models have demonstrated utility in assigning a specific donor to a list of potential recipients without compromising the principles of urgency and utility [230]. Meanwhile, other models have demonstrated excellent diagnostic accuracy in predicting de novo or recurrent liver disease, the development of significant fibrosis, recurrent HCC, the onset of MetS, or renal damage in the post-LT setting [228]. Further studies need to be conducted in this field to enhance personalized medicine for these patients. Finally, it will be essential to continue fostering clinical studies and translational research to deepen our understanding of the immunological mechanisms underlying immunosuppression and immunotolerance.

## Data Availability

No new data were created.
